# Liver Disease and Other Comorbidities in Wolcott-Rallison Syndrome: Different Phenotype and Variable Associations in a Large Cohort

**DOI:** 10.1159/000369804

**Published:** 2015-02-05

**Authors:** Abdelhadi M. Habeb, Asma Deeb, Matthew Johnson, Mohammed Abdullah, Majidah Abdulrasoul, Hussain Al-Awneh, Mohammed S.F. Al-Maghamsi, Fathiya Al-Murshedi, Ramlah Al-Saif, Siham Al-Sinani, Dina Ramadan, Hala Tfayli, Sarah E. Flanagan, Sian Ellard

**Affiliations:** ^a^Paediatric Department, Prince Mohammed bin-Abdulaziz Hospital, Madinah, UK; ^b^Endocrine and Diabetes Unit, Maternity and Children Hospital, Madinah, UK; ^c^Paediatric Department, Maternity and Children Hospital, Dammam, Saudi Arabia, UK; ^d^Paediatric Endocrinology Department, Mafraq Hospital, AbuDhabi, United Arab Emirates, UK; ^e^Institute of Biomedical and Clinical Science, University of Exeter Medical School, Exeter, UK; ^f^Paediatric Department, Khartoum University, Khartoum, Sudan; ^g^Paediatric Department, Kuwait University, Kuwait; ^h^Paediatric Department, Sabah Hospital, Kuwait; ^i^Paediatric Endocrinology Division, Queen Rania Al Abdullah Hospital for Children, KHMC, RMS, Amman, Jordan; ^j^Genetic and Developmental Medicine Clinic, Muscat, Oman; ^k^Gastroenterology Unit, Department of Child Health, Sultan Qaboos University Hospital, Muscat, Oman; ^l^Department of Pediatrics and Adolescent Medicine, American University of Beirut Medical Center, Beirut, Lebanon

**Keywords:** *EIF2AK3* mutations, Hepatitis, Childhood diabetes, Liver transplantation, Skeletal dysplasia

## Abstract

**Background:**

Wolcott-Rallison syndrome (WRS) is caused by recessive *EIF2AK3* mutations and characterized by early-onset diabetes and skeletal dysplasia. Hepatic dysfunction has been reported in 60% of patients.

**Aims:**

To describe a cohort of WRS patients and discuss the pattern and management of their liver disease.

**Methods:**

Detailed phenotyping and direct sequencing of *EIF2AK3* gene were conducted in all patients.

**Results:**

Twenty-eight genetically confirmed patients (67% male; mean age 4.6 years) were identified. 17 different *EIF2AK3* mutations were detected, of which 2 were novel. The p.S991N mutation was associated with prolonged survival and p.I650T with delayed onset. All patients presented before 25 months with diabetes with variation in the frequency and severity of 10 other features. Liver disease, first manifested as non-autoimmune hepatitis, was the commonest extra-pancreatic feature identified in 85.7% (24/28). 22/24 had at least one episode of acute hepatic failure which was the cause of death in all deceased patients (13/28). One child was treated by liver transplantation and had no liver disease and better diabetes control for the following 6 years.

**Conclusions:**

Liver disease in WRS is more frequent than previously described and carries high mortality. The first experience with liver transplantation in WRS is encouraging.

## Introduction

Wolcott-Rallison syndrome (WRS) is a rare condition that was initially described in 1972 in siblings with early-onset diabetes mellitus and skeletal dysplasia (SD) [1]. Further reports extended the phenotype to recurrent hepatitis, renal dysfunction, failure to thrive, developmental delay, neutropenia and hypothyroidism [2,3,4,5,6,7]. Most reported families with WRS originate from the Middle East [8,9], and the condition is the commonest cause of permanent neonatal diabetes mellitus (PNDM) in consanguineous pedigrees [4] and Arabs [10].

WRS is caused by recessive loss of function mutations in the *EIF2AK3* gene [11], and almost all reported cases have identifiable mutations. The *EIF2AK3* gene encodes a protein called pancreatic PKR-like endoplasmic reticulum kinase (PERK), which plays a key role in detecting and initiating the cellular response to endoplasmic reticulum stress. Failure of appropriate PERK response results in accumulation of misfolded proteins, which leads to cell damage and apoptosis [12,13].

Liver disease was reported in some patients with WRS since 1982 [2,3,4,5,6,7,14]. The typical manifestation was intermittent hepatitis precipitated by stress and characterized by raised liver transaminase, jaundice and hepatomegaly. Acute fatal hepatic failure and chronic hepatic dysfunction were also reported [8]. The hepatic histology varied from progressive fibrosis with mild steatosis and intrahepatic cholestasis [2] to preserved architecture with mild lobular infiltration by lymphocytes and oedema of the portal spaces [15]. The frequency of liver disease was 60% in 35 WRS patients reviewed by Ozbek et al. [5] in 2009. However, our clinical impression was that liver disease in WRS is more frequent and has high mortality. The aim of this study was to describe a new cohort of patients with WRS and discuss the pattern and management of hepatic dysfunction in this cohort.

## Patients and Methods

The study was conducted according to the Declaration of Helsinki.

We searched the database at the Exeter molecular genetics laboratory for genetically confirmed WRS cases referred from Arab states until July 2014. Paediatricians in the region were contacted to identify unreported WRS cases tested outside Exeter. A questionnaire on the details of the genotype and phenotype of WRS was distributed to the referring physicians of unreported cases and they were also requested to provide follow-up data of their reported WRS cases. We excluded patients in whom clinical data were incomplete or if their clinicians did not return the questionnaire. We defined liver disease as the presence of at least one episode of non-infective non-autoimmune hepatitis, unexplained jaundice, hepatomegaly, high liver transaminase, deranged liver function or acute hepatic failure (AHF; INR of >1.5 and evidence of impaired sensorium). The frequency of liver disease in WRS was expressed as the percentage of patients with hepatic dysfunction of the total number of the studied cohort.

Informed consent was signed by the parents, and DNA was extracted from the whole blood using the standard methods. Patients were tested for mutations in the *KCNJ11, ABCC8 INS* and *EIF2AK3* genes by Sanger sequence analysis as previously described [4].

## Results

In total, 32 patients were identified. Four patients were excluded from the analysis (2 with incomplete follow-up data and 2 because their physicians did not return the questionnaire). 28 patients (20 families) from 8 Arab countries were studied. Of these, 18 were not previously described and 10 were initially reported by us in 2009 and 2011, but remained under follow-up by the same physicians since diagnosis. The genotypes and phenotypes of studied patients are shown in table 1.

### Clinical Characteristics

**Fig. 1:**
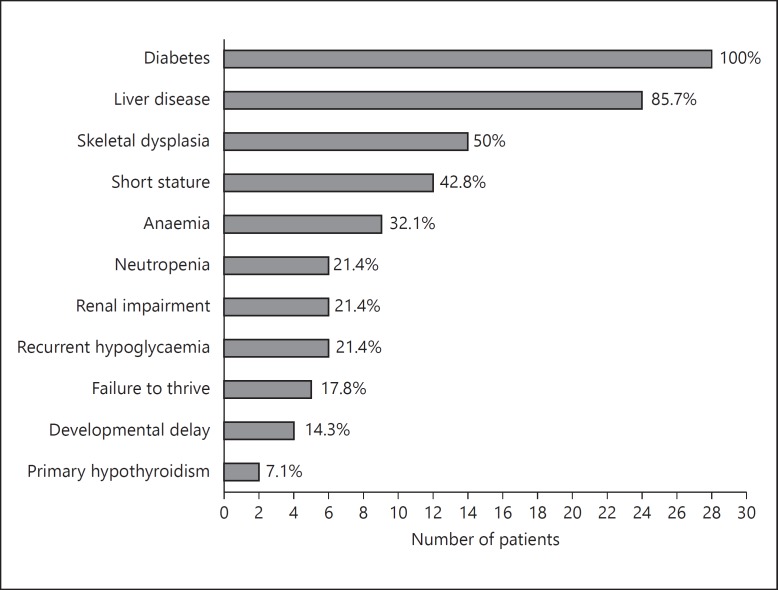
Frequency of clinical features of WRS in 28 Arab patients expressed as number and percentage of the total patients (features reported once are not included).

All patients were the product of consanguineous marriages; their mean age was 4.6 years (range: 10 months to 17.5 years) and 67% were male. There was a variation in the phenotype between patients with the same mutations including siblings. Diabetes was the presenting feature in all subjects: 25/28 patients have PNDM (onset <6 months old; mean age at diagnosis 7.6 weeks) and 3 presented at 14, 18 and 24 months old. 46.4% of patients were deceased (13/28) at a mean age of 5.8 years, and all living subjects were on insulin therapy. The longest survived patient in the cohort was 17.5 years. Two patients have isolated PNDM, and the rest showed at least one extra-pancreatic feature (fig. 1).

### Frequency and Pattern of Liver Disease

Twenty-four of 28 patients have liver disease, giving a frequency rate of 85.7%. The mortality rate in patients with liver disease was 54.1% (13/24), and AHF was the cause of death in all deceased subjects. During follow-up of the 10 reported cases, 2 experienced their first hepatitis episode and 3 died of AHF. The first presentation of liver disease in all 24 patients was acute non-autoimmune hepatitis triggered by viral illnesses and characterized by high liver enzymes, jaundice and hepatomegaly. In 6 patients, the hepatitis episodes were associated with impaired renal function (table 1). Seven patients had hepatitis at diagnosis, along with diabetes, and the remaining 17 patients experienced their first episode between 2 weeks and 2 years after the onset of diabetes. In 3 children, the first hepatitis episode proceeded to fatal AHF; however, in the remaining 21 patients the episodes were intermittent, lasting for 3-20 days, with full recovery except in patient 11.1 who continued to have persistent hepatomegaly. The highest liver transaminase levels during the episodes varied between patients from 242 to 50,000 IU/l (mean 8,620). 22/24 patients experienced at least one episode of AHF. Of these, 13 died, 8 recovered with conservative management and 1 child was treated by liver transplantation (LT). This child (13.1) was diagnosed with diabetes at 14 months old and needed LT at 28 months to treat his first AHF. The genetic diagnosis of WRS was subsequently confirmed. During a 6-year post-transplant follow-up, he maintained normal liver function without hepatitis, and his average HbA1c was 7.8%. He remained short due to marked hip and knee deformities; however, his average growth velocity over the last 3 years was 6 cm/year (online suppl. fig. S1; for all online suppl. material, see www.karger.com/doi/10.1159/000369804). Liver biopsy was performed between hepatitis episodes in 4 patients. In 12.1, 2 and 6, it was for a second opinion and in patient 11.1 for persistent hepatomegaly before the genetic testing was conducted. The histological features were variable between the 4 patients including markedly swollen hepatocytes with rarefied cytoplasm, occasional necrotichepatocytes, minimal fibrosis and cholangiopathy.

### Genotype

Genetic diagnosis was confirmed in all 28 patients (table 1). 25 patients were tested in Exeter, while patients 6.1, 7.1, and 11.1 were tested elsewhere, and the results were provided by their physicians. 17 different homozygous *EIF2AK3* mutations were identified of which 2, p.S991N and p.G1010D, were novel. Both mutations affect residues that are highly conserved across species, and in silico analysis predicts that both substitutions are disease causing (Alamut Interactive Biosoftware, version 1.5, Rouen, France). The most frequent mutation, p.V349Sfs*3 (c.1044_1057del), was detected in 3 families followed by p.W430X (c.1290G>A) identified in 2 families (6 patients from an extended family; online suppl. fig. S2). There was no genotype-phenotype correlation apart from longer survival of 17.5 years associated with the p.S991N mutation, and a delayed age at onset of 14 months with the p.I650T mutation.

## Discussion

We studied the genotype and phenotype of 28 patients with WRS from 8 Arab countries. Two novel *EIF2AK3* mutations and 18 new patients were described. This is the first study to focus on liver disease in WRS, and it also provides long-term data on the first child with WRS to undergo LT.

All patients presented with diabetes, and liver disease was the commonest extra-pancreatic feature and the cause of death in all deceased children. The frequency of hepatic disease in our cohort was 85% compared to 60% reported by Ozbek et al. [5]. The most likely explanation for the higher frequency of liver disease in our cohort is the availability of long-term follow-up data on some of our patients. Of the 10 reported patients in this series, 2 experienced their first hepatitis episode after the initial report and 3 died of AHF during follow-up. Of note, our patients without liver disease were younger than 3.5 years. Considering the disease course in this cohort, we suspect that all of them might eventually develop liver disease.

Children with WRS typically present in the first few months of life with diabetes, and it is recommended that any child of consanguineous parents presenting with diabetes within the first 6 months of life should be tested for *EIF2AK3* mutations [4]. All our patients presented with diabetes; however, 3 of them were diagnosed at 14, 18 and 24 months, making a total number of 4 WRS cases with delayed onset reported to date [3]. We suggest that WRS should be considered in children of consanguineous families diagnosed with diabetes within the first 2 years of life. Of the 4 mutations detected in patients with delayed presentation, the p.N656K and p.I650T missense mutations were only reported in these patients and appear to be associated with delayed onset of WRS. Both were located close to each other on the first kinase domain of PERK protein residue, and the p.N656K mutation was shown to have a residual kinase activity, which may explain the delayed onset in that patient [3]. However, the other 2 mutations (p.W164X and p.N42Tfs*14) were reported in other patients with early-onset WRS [3,4,16]. The mean survival age in our cohort was 5.8 years, which is similar to the figure reported by Ozbek et al. [5]. However, a mild course and prolonged survival of 32 and 35 years was reported in 2 patients with the missense mutations p.F593 and p.L646, respectively [3,4,14]. Interestingly, our longest surviving patient of 17.5 years has mild liver disease and was homozygous for a novel missense mutation (p.S991N). It is possible that these 3 missense mutations are associated with residual kinase activity of the PERK protein resulting in a mild phenotype and prolonged survival; however, functional studies are required to clarify this hypothesis.

In agreement with previous reports [3,4,16,17], we found a variation in the phenotype between patients with the same mutation. The exact cause of this phenomenon is still unclear; however, it seems likely that other genetic and environmental factors influence the tissue response to the mutant PERK protein. Primary hypothyroidism (PH) has been recently reported in 3 patients with WRS [4,18,19]. We add 2 further patients (7.1 and 17.1) with PH, supporting the suggestion that PH is a new feature of this syndrome. Recurrent hypoglycaemia was occasionally described in WRS [20,21], and studies in *Perk* knockout mice suggested a role of impaired hepatic gluconeogenesis [22]. Despite the high frequency of liver disease in our cohort, symptomatic hypoglycaemia was only documented in 6 patients with liver disease from a family with a p.W430X mutation. The only child with this mutation without recurrent hypoglycaemia was from a different family and experienced just one episode of hepatitis. It is possible that the p.W430X mutation is linked to recurrent hypoglycaemia in patients with frequent hepatitis, or the family has another predisposing factor for hypoglycaemia. SD is an essential criteria of WRS; however, its frequency in our cohort was lower than previously described [2,5]. It is possible that some of our patients were still too young to develop it or have no regular skeletal survey to detect subtle skeletal changes.

All our patients with liver disease presented with non-autoimmune hepatitis triggered by viral illnesses, and most of them recovered completely from the initial episodes. However, in 3 children the first hepatitis attack proceeded to fatal AHF. This indicates that the course of WRS-related hepatitis is unpredictable and that every episode should be considered as potentially fatal. WRS-related hepatitis usually manifest after the onset of diabetes; however, Engelmann et al. [23] described a child with WRS in whom the first hepatitis episode developed a few months before diabetes, and 25% of our patients had hepatitis at initial diagnosis along with diabetes. The exact mechanism of liver dysfunction in WRS is unknown; however, the fact that the episodes are triggered by viral illness and other stresses suggest the inability of liver cells to deal with endoplasmic reticulum stress due to *EIF2AK3* mutations. We noticed a variation in the age at onset, frequency, and prognosis of hepatitis between patients. The fact that this variation was documented in 7 children with the p.W430X mutation and 5 patients with the p.V349Sfs*3 suggests that the severity of liver disease is not related to the specific mutation. In this series, the prognosis of acute liver failure was not related to the age, gender or the number of hepatitis episodes (results not shown). However, the retrospective nature of our study limited our ability to define the role of other clinical or laboratory variables in the prognosis of liver disease in WRS. The histological findings in our 4 patients who had liver biopsy were also variable even between children with the same mutation. Although liver biopsy may be useful in understanding the mechanism of liver disease, given the availability of genetic testing, we feel that its clinical value in WRS is very limited.

The management of liver disease in WRS is a challenge as the mortality is high and the course is unpredictable. Our practice is to ensure that families recognize the symptoms of hepatitis, and we request parents to bring the child to hospital for possible admission if he/she develops flu-like illness or other stress. We prefer early assessment in a tertiary care liver unit, so a care plan is prepared to avoid any delay in starting the management of AHF. Confirming the genetic diagnosis, particularly in patients with first hepatitis episodes or atypical features, would avoid the need for invasive tests such as liver biopsy. LT has been successfully used in some children with hereditary disorders [24]; however, to the best of our knowledge, its use in WRS has been limited to 2 children: the first one was our patient 11.1, and the second was a 6-year-old girl described in a recent media report [25]. Our patient had the transplantation at 2.4 years of age following his first AHF, and the diagnosis of WRS was not confirmed at that time. The LT saved his life and appeared to cure the liver disease. The improvement of his diabetes control is unlikely to be directly related to the LT, but may be reflecting a better lifestyle with no recurrent hepatitis. His SD led to severe hip and knee deformities, explaining his short stature; however, his recent growth was comparable to other children with liver transplant [26]. Our experience with this child suggests that LT would be a successful therapy for WRS patients; however, more data are needed to make it a standard treatment for WRS. As WRS affects other organs such as pancreatic β-cell and kidneys, combined organ transplant may be an option for these patients. Clearly, the decision of transplantation should take into account the neurocognitive function of the child as some WRS patients have significant neurodevelopmental delay.

In conclusion, WRS should be considered in children of consanguineous families diagnosed with diabetes within the first 2 years of life. In this condition, liver disease is more common than previously reported, has unpredictable course and carries high mortality. The first experience of LT in WRS is encouraging.

## Supplementary Material

Supplementary data

## Figures and Tables

**Table 1:** Demography, genotype and clinical characteristics of the 28 studied patients with WRS

Patient	Country of origin	Gender	Age at diagnosis and presenting features	EIF2AK3 mutation	Acute hepatitis – age at 1st episode and frequency	Acute hepatic failure –age at onset, number, and outcomes	Other features	Prognosis/comments
1.1	KSA	Male	Hyperglycaemia at 8 weeks	p.W430X (c.1290G>A)	1 episode at 8 months	No	No	Alive at 18 months old

2.1 [17]	KSA	Male	DKA at 21 weeks	p.V349Sfs*3 (c.l044_1057del)	20 months; 8 episodes (first after reporting)	1 fatal episode at 6 years	SD, SS, FTT and impaired RF during hepatitis	Died at 6 years

3.1 [4, 17]	KSA	Female	DKA and hepatitis at 8 weeks	p.V349Sfs*3 (c.l044_1057del)	8 weeks; 20 episodes	2 episodes at 3 years and 1 fatal at 7.5 years	No	Died at 7.5 years (after initial reporting)

3.2 [17]	KSA	Female	DKA and hepatitis at 10 weeks	p.V349Sfs*3 (c.l044_1057del)	10 weeks; 8 episodes	1 fatal episode at 2 years	SD, SS and impaired RF during hepatitis	Died at 2 years

3.3 [17]	KSA	Male	Hyperglycaemia at 6 weeks	p.V349Sfs*3 (c.l044_1057del)	2 years; once proceeded to acute hepatic failure	1 fatal episode at 2 years	No	Died at 2 years

4.1 [17]	KSA	Male	DKA at 8 weeks	p.V349Sfs*3 (c.l044_1057del)	18 months; 4 episodes (first after reporting)	1 episode (after first reporting)	No	Alive at 6 years

5.1^a^	KSA	Male	Hyperglycaemia at 2 years	P.W163X (c.491G>A)	2.4 years; once proceeded to acute hepatic failure	1 treated conservatively	Impaired RF during hepatitis episode	Alive at 5 years

6.1^a^	KSA	Male	Hyperglycaemia at 1.5 years	p.N420Tfs*14 (c.l259del)	2.5 years; once proceeded to hepatic failure	1 fatal episode at 7 years	SD, SS and impaired RF during hepatitis	Died at 7 years

7.1	Jordan	Male	DKA and hepatitis at 6 weeks	p.G1010D (c.3029G>A)	6 weeks; 4 episodes	1 fatal episode at 3.5 years	SD, SS, PH, ASD, DD and epilepsy	Died at 3.5 years

8.1^a^	Kuwait	Male	Hyperglycaemia at 15 days	FS 523STOP (delGAAA1639-42)	10 months; once proceeded to acute hepatic failure	1 fatal episode at 10.5 months	Impaired RF during hepatitis episode	Died at 10.5 months

9.1	Iraq	Male	Hyperglycaemia at 7 weeks	p.W520X (c.1560G>A)	No	No	No	Alive at 14 months; siblings died of WRS

10.1	Lebanon	Female	DKA at 11 weeks	p.R1064X (c.3190C>T)	11 months; 1 episode recovered spontaneously	No	Impaired RF during hepatitis and deafness	Alive at 15 months; siblings died of WRS

11.1	Oman	Male	Hyperglycaemia at 4 weeks	P.Y588X (c.1764T>G)	10 months; 6 episodes	2 episodes, 1 led to death	Neutropenia, SD, SS, FTT, DD and ADHD	Died at 6.5 years; had liver biopsy for persistent hepatomegaly

11.2	Oman	Female	DKA and hepatitis at 6 weeks	P.Y588X (c.1764T>G)	6 weeks; 5 episodes	1 treated conservatively	Neutropenia and FTT	Alive at 5 years

12.1	UAE	Female	DKA at 6 weeks	p.W430X (c.!290G>A)	8 weeks; 9 episodes	1 treated conservatively	Anaemia and RH	Alive at 10 months; had liver biopsy

12.2	UAE	Female	DKA at 10 weeks	p.W430X (c.1290G>A)	11 weeks; 7 episodes	4 episodes treated conservatively	SD, SS anaemia, neutropenia, squint and RH	Alive at 2.5 years; had liver biopsy

12.3 [4]	UAE	Male	DKA and hepatitis at 7 weeks	p.W430X (c.1290G>A)	7 weeks; 7 further episodes	1 fatal episode	SD, SS, anaemia, neutropenia and RH	Died at 7 years

12.4 [4]	UAE	Male	DKA and hepatitis at 6 weeks	p.W430X (c.1290G>A)	6 weeks; >8 episodes	3 episodes, 1 resulted in death	SD, SS, anaemia, neutropenia and RH	Died at 4 years

12.5 [4]	UAE	Male	DKA at 6 weeks	p.W430X (c.1290G>A)	8 weeks; once/3 months	6 episodes, 1 resulted in death	SD, SS, anaemia, neutropenia and RH	Died at 5 years

12.6	UAE	Female	Hyperglycaemia and hepatitis at 7 weeks	p.W430X (c.1290G>A)	7 weeks; 6 episodes	4 episodes treated conservatively	Pancytopenia, FTT and RH	Alive at 2.4 years; had liver biopsy

13.1 [4]	UAE	Male	Hyperglycaemia at 14 months	P.I650T (c.1949T>C)	1 episode at 2 years; none since transplant	1 episode led to transplant	Anaemia, SD and SS	Alive at 8 years

14.1 [4]	UAE	Male	DKA at 8 weeks	P.G956E (c.2867G>A)	10 weeks; >15 episodes	1 fatal episode (after reporting)	SD, SS and anaemia	Died at 15 years (after initial reporting)
15.1	UAE	Male	Hyperglycaemia at 8 weeks	P.E524X (c.l567_1570del)	9 months; 3 episodes	1 episode treated conservatively	SD and anaemia	Alive at 18 months

16.1	UAE	Female	DKA at 6 weeks	p.? (c.1427-?_2490+?del)	8 months; 2 episodes	1 fatal episode	No	Died at 2.9 years

17.1	Kuwait	Male	Hyperglycaemia at 2 months	P.S991N (c.2972G>A)	14 months; 2 episodes	2 episodes treated conservatively	DD, SD, SS and PH	Alive at 17.5 years

18.1	Sudan	Male	Hyperglycaemia, at 2 months	p.P269fs (c.802_803dup)	No	No	DD and SD	Alive at 3.5 years

19.1	Sudan	Female	Hyperglycaemia at 2 months	P.Y989X (c.2967T>A)	No	No	No	Alive at 3 years; twin brother healthy

20.1	Sudan	Male	Hyperglycaemia at 2 months	p.? (c.1647+2T>A)	No	No	FTT	Alive at 2 years

All mutations listed are homozygous. KSA = Kingdom of Saudi Arabia; UAE = United Arab Emirates; DKA = diabetic ketoacidosis; FTT = failure to thrive; SS = short stature; RF = renal function; ADHD = attention deficit hyperactivity disorder; ASD = atrial septal defect; RH = recurrent hypoglycaemia; DD = developmental delay. All nucleotide and amino acid numbering refers to transcript AF110146.1, which contains 7 leucine residues in the microsatellite region of exon one. The c.l427-?_2490+?del and c.1647+2T>A mutations have been listed at the protein level as p.?, which denotes that the protein has not been analyzed, although an effect is expected.

a These samples were tested outside Exeter.
