# Supplementary material for: Liver Disease and Other Comorbidities in Wolcott-Rallison Syndrome: Different Phenotype and Variable Associations in a Large Cohort
Source: Horm Res Paediatr. 2015 Feb 5;83(3):190–7. doi: 10.1159/000369804 (PMC4464042; doi:10.1159/000369804)
Supplement: Supplementary file 1 — Supplementary data [file hrp-0083-0190-s01.doc]

Supplement figure S1: growth chart of the first child with Wolcott Rallison syndrome to undergo liver transplantation

Supplement figure S2: pedigree of family 12 with the p.W430X mutation
